# Visceral adiposity index and atherogenic index of plasma as useful predictors of risk of cardiovascular diseases: evidence from a cohort study in Iran

**DOI:** 10.1186/s12944-021-01505-w

**Published:** 2021-08-01

**Authors:** Behrooz Hamzeh, Yahya Pasdar, Narmin Mirzaei, Roya Safari Faramani, Farid Najafi, Ebrahim Shakiba, Mitra Darbandi

**Affiliations:** 1 Research Center for Environmental Determinants of Health (RCEDH), Health Institute, Kermanshah University of Medical Sciences, Kermanshah, Iran; 2 Social Development and Health Promotion Research Center, Kermanshah University of Medical Sciences, Kermanshah, Iran; 3 Cardiovascular Research Center, Kermanshah University of Medical Sciences, Kermanshah, Iran; 4 Student Research Committee, Kermanshah University of Medical Sciences, Kermanshah, Iran

**Keywords:** Cardiovascular Diseases, Body Mass Index, Primary Prevention, Cholesterol, Obesity, Persian

## Abstract

**Background:**

Visceral adiposity index (VAI) and atherogenic index of plasma (AIP) are relatively new indicators for predicting non-communicable diseases (NCDs). Therefore, the present study was done to assess the association of AIP and VAI with risk of cardiovascular diseases (CVDs).

**Methods:**

This cross-sectional study was conducted on 7,362 individuals aged between 35 and 65 years old participated in Ravansar non-communicable diseases (RaNCD) cohort study. AIP was calculated based on levels of triglyceride and high -density lipoprotein cholesterol (HDL-C). VAI was calculated using values of body mass index (BMI), waist circumference (WC), triglyceride, and HDL-C. Logistic regression models were used to assess the association of AIP and VAI with risk of CVDs.

**Results:**

Mean values of anthropometric indices, lipid profile, AIP, and VAI were significantly higher in patients with CVDs than individuals without CVDs (*P* < 0.001). Mean values of anthropometric indices, lipid profile, and NCDs including hypertension, dyslipidemia, diabetes, metabolic syndrome (MetS), and CVDs in the third tertile of AIP and VAI were significantly increased compared to the first tertile (*P* < 0.001). After adjusting confounding factors, risk of CVDs in the third tertile of AIP was (OR = 1.32, 95 % CI: 1.03, 1.69) significantly increased compared to the first tertile. Risk of CVDs in the third tertile of VAI was (OR = 1.48, 95 % CI: 1.12, 1.97) significantly increased compared to the first tertile.

**Conclusions:**

According to the findings, AIP and VAI were positively associated with risk of CVDs. Therefore, AIP and VAI can be useful in identifying high-risk subgroups of CVDs in general population.

## Background

Cardiovascular diseases (CVDs) are the main cause of death worldwide. So that, 32 % of all deaths in the world have been estimated to be due to CVDs in 2019. More than 75 % of CVDs-related deaths occur in low- and middle-income countries [1]. Approximately 50 % of annual deaths and 50 % of deaths caused by non-communicable diseases (NCDs) are due to CVDs in Iran [2]. Obesity and dyslipidemia are the known risk factors for CVDs, which are easily preventable and changeable [3–5]. Therefore, their screening can be useful for prediction and early detection of CVDs in populations.

In a meta-analysis study (2020), simple anthropometric indices including waist circumference (WC), body mass index (BMI), and waist to hip ratio (WHR) were introduced as acceptable predictors of CVDs [6]. However, these indices cannot measure visceral and subcutaneous fat, while visceral adipose tissue (VAT) and subcutaneous adipose tissue (SAT) have an important role in pathogenesis of CVDs [7, 8]. VAT can be evaluated using abdominal MRI (magnetic resonance imaging) or computed tomography (CT) scan; but these methods are expensive and limited and are not used for screening in large populations [8]. The visceral adiposity index (VAI) is a mathematical model to estimate VAT, combining anthropometric (WC and BMI) and laboratory parameters (triglyceride (TG) and high-density lipoprotein cholesterol (HDL-C)) [9]. Previous studies have reported validity of VAI for predicting NCDs including metabolic syndrome (MetS), type 2 diabetes mellitus (T2DM), and hypertension [10–12]. Previous studies have also demonstrated a mean VAI between 2.1 and 3.2 in the Iranian population [13–15].

Atherogenic dyslipidemia is characterized by an increase in levels of TG and low-density lipoprotein cholesterol (LDL-C) and a decrease in level of HDL-C in the blood, which has been identified as an important marker of CVDs [16, 17]. The atherogenic index of plasma (AIP), which is logarithmic conversion of TG into HDL-C ratio, has been introduced as a strong predictor of atherosclerosis and CVDs in some populations [18]. AIP is correlated with lipoprotein particle size of LDL-C, HDL-C, and very-low-density lipoprotein (VLDL) acting as one of the most sensitive markers of CVDs [19–21], and mean level of AIP has been reported between 0.17 and 0.41 in the Iranian populations [20, 22].

To the best of knowledge, there is no study assessed the association between AIP and VAI with risk of CVDs in Kurdish ethnicity so far. Therefore, this study was done to assess the association of AIP and VAI with CVDs in adults with Kurdish ethnicity, using the Iranian Ravansar non-communicable diseases (RaNCD) cohort data.

## Methods

### Study design and participants

This cross-sectional study was conducted using data from RaNCD cohort study. The RaNCD cohort study is a part of the prospective epidemiological research studies in Iranian adults (PERSIAN) cohort. In the PERSIAN cohort, all 19 cohort sites (covering a representative sample of different Iranian ethnicities) used the same questionnaire and aimed to follow up all participants for the next 15 years. Further information is available at the following address (http://persiancohort.com). Ravansar is a district with a population of about 50,000 people, located in western Iran and in Kermanshah Province. The number of participants in baseline phase of the RaNCD was equal to 10,047 adults, all of whom were permanent residents of Ravansar. Details of the RaNCD methodology have been described elsewhere [23].

### Inclusion and exclusion criteria

**Fig. 1:**
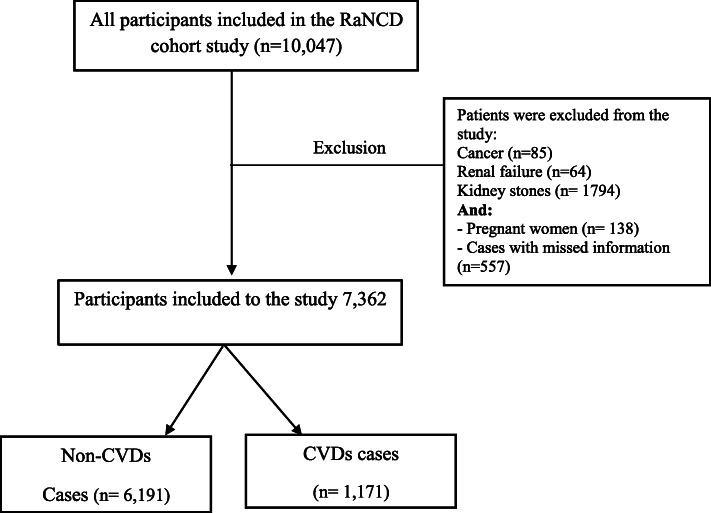
Flow chart of study

All subjects enrolled in baseline phase of RaNCD prospective study entered the present study (*n* = 10,047). The subjects with cancer (*n* = 85), renal failure (*n* = 64), kidney stones (*n* = 1,794), pregnant woman (*n* = 138) and cases with the missed information (*n* = 557) were excluded from the study, finally 7,362 subjects remained to be included in the present study (Fig. 1).

### Data collection

Questionnaire information was completed by experts of the cohort center through face-to-face interviews. Demographic information including age, sex, smoking, and history of chronic diseases was recorded online in an electronic data collection form. Biochemical parameters, anthropometric indices, and blood pressure level were measured according to the PERSIAN cohort protocol.

### Physical activity

Physical activity was measured using metabolic equivalent rates (METs) of self-reported daily activities of participants in PERSIAN cohort using the questionnaire consisting of 22 questions about their sport, work, and leisure- related activities on an average weekday. The questionnaire information was extracted and used based on Met/ hour per week. [24].

### Blood pressure measurements

Blood pressure was measured using a manual sphygmomanometer (Riester) from both arms, in sitting position and after 10 min of rest, and its mean was reported. Hypertension was defined as having systolic blood pressure (SBP) ≥ 140 and diastolic blood pressure (DBP) ≥ 90 or the current use of medication for hypertension [25].

### Anthropometric measurements

Body weight was measured using bioelectrical impedance analyzer (BIA) (Inbody 770, Inbody Co, Seoul, Korea) with a precision of 0.5 kg. Other anthropometric measurements including body fat mass (BFM), percentage of body fat (PBF), skeletal muscle mass (SMM), and visceral fat area (VFA) were also done using BIA. Height of the participants was measured by BSM 370 (Biospace Co, Seoul, Korea) with a precision of 0.1 cm. WC was measured with a flexible measuring tape at the midpoint between lower rib margin and the iliac crest to the nearest 0.5 cm. BMI was calculated by the following formula: weight (kg) divided by square of height (m). VAI was also calculated by the following formula [9]:

$$ \mathrm{Males}:\mathrm{VAI}=\left(\frac{WC\ (cm)}{39.68+\left(1.88\times BMI\ \left( kg/m2\right)\right)}\right)\times \left(\frac{TG\ \left( mmol/l\right)}{1.03}\right)\times \left(\frac{1.31}{HDL\ \left( mmol/l\right)}\right) $$

$$ \mathrm{Females}:\mathrm{VAI}=\left(\frac{WC\ (cm)}{39.58+\left(1.89\times BMI\ \left( kg/m2\right)\right)}\right)\times \left(\frac{TG\ \left( mmol/l\right)}{0.81}\right)\times \left(\frac{1.52}{HDL\ \left( mmol/l\right)}\right) $$

After performing analyses of this study, VAI tertiles (< 2.4; 2.4–4.5; >4.5) were also extracted for the study sample [26].

### Biochemical measurements

Blood samples were collected after 8–12 h fasting to measure biochemical markers including lipid profile (TG, LDL-C, HDL-C, and total cholesterol (TC)), fasting blood sugar (FBS), and liver enzymes like alkaline phosphatase *(*ALP*)*, aspartate transaminase (AST), alanine aminotransferase (ALT), and gamma-glutamyl transferase (GGT). AIP was calculated using the following formula: log_10_ (TG/HDL-C) [21]; which can be classified based on the obtained values as follows: < 0.11 for low risk, 0.11–0.24 for moderate, and 0.24 < for high risk of CVDs [22].

Dyslipidemia was defined as having LDL-C ≥ 160 mg/dL and/or TC ≥ 240 mg/dl and/ or HDL- C < 40 mg/dl and/or TG ≥ 200 mg/dl and/or a history of taking medication for this condition [25]. MetS was defined according to the international diabetes federation (IDF) criteria [27].

### Definition of outcome

Participants who had at least one of the following conditions were considered as patients with CVDs: A history of ischemic heart disease (IHD), heart failure and angina, stroke, myocardial infarction (MI) and/or the current use of medication for CVDs. The definition of CVDs is based on the international statistical classification of diseases and related health problems (ICD10) (10th revision). Type of CVDs was determined based on diagnosis of a cardiologist, then was classified using the ICD10 code.

### Data analysis

Data analysis was performed using Stata software version 14.1 (Stata Corp, College Station, TX, USA). In descriptive reports, quantitative variables were presented as mean ± standard deviation or median (interquartile range = IQR), and qualitative variables were expressed as frequency (percentage). Baseline characteristics of the studied participants were compared by Chi-Square and t-test or Mann Whitney U test between CVDs and non-CVDs groups. One-way analysis of variance (ANOVA) test was used to compare variables in AIP and VAI tertiles. According to binary outcome (CVDs), simple and multiple logistic regression models were applied to assess the association between AIP and VAI with CVD, and variables with *P-*value < 0.2 in univariate analysis were entered into multivariable logistic model [28]. The crude and adjusted odds ratios with 95 % confidence interval were reported. The *P*- value < 0.05 was considered to be statistically significant in all statistical tests.

## Results

### Characteristics of the participants

Finally, 7,362 subjects out of 10,047 participants of RaNCD were eligible for data analysis. The baseline demographic, biochemical, and anthropometric characteristics of the participants are shown in Table 1. Mean age of the participants was equal to 47.20 ± 8.33 years old and 3,420 (46.45 %) subjects were male. Mean VAI for participants with CVDs was significantly higher compared to participants without CVDs (*P* < 0.001). Median (IQR) of AIP in CVDs and non-CVDs groups was equal to 0.09 (-0.33, 0.55) and 0.24 (-0.17, 0.62), respectively (*P* < 0.001). Other anthropometric indices, TG, LDL-C, TC, FBS, ALP, and GGT were significantly higher in patients with CVDs compared to those without CVDs. Prevalence of MetS in the CVDs group (53.80 %) was significantly higher than the non-CVDs group (20.37 %), (*P* < 0.001).

**Table 1:** Baseline characteristics according to cardiovascular diseases (CVDs) status

Parameters	Total (*n* = 7362)	Non-CVDs (*n* = 6191)	CVDs (*n* = 1171)	*P* value*
Gender, n (%)				
Male	3420 (46.45)	3036 (49.04)	384 (32.79)	< 0.001
Female	3942 (35.55)	3155 (50.96)	787 (67.21)	
Age (year)	47.20 ± 8.33	46.03 ± 7.90	53.39 ± 7.79	< 0.001
Current smoker, n (%)	865 (22.33)	764 (23.45)	101 (16.40)	< 0.001
Physical activity, n (%)				
Low	2168 (29.45)	1736 (28.04)	432 (36.89)	< 0.001
Moderate	3539 (48.07)	2979 (48.12)	560 (47.82)	
Vigorous	1655 (22.48)	1476 (23.84)	179 (15.29)	
BMI (kg/m^2^)	27.44 ± 4.67	27.20 ± 4.63	28.69 ± 4.66	< 0.001
WHR	0.94 ± 0.06	0.93 ± 0.06	0.95 ± 0.06	< 0.001
WC (cm)	96.92 ± 10.55	96.28 ± 10.43	100.30 ± 10.59	< 0.001
BFM (kg)	24.99 ± 9.62	24.42 ± 9.54	27.99 ± 9.45	< 0.001
PBF	33.82 ± 9.58	33.09 ± 9.56	37.69 ± 8.73	< 0.001
VFA (cm^2^)	121.93 ± 51.83	118.18 ± 51.29	141.71 ± 50.13	< 0.001
SLM (kg)	3085 (41.90)	45.49 ± 9.07	42.82 ± 8.40	< 0.001
VAI (male)	1.93 ± 1.50	1.90 ± 1.48	2.11 ± 1.57	< 0.001
VAI (female)	2.82 ± 2.10	2.77 ± 2.10	3.10 ± 2.20	< 0.001
AIP^*^	0.11 (-0.29, 0.56)	0. 0.09 (-0.33, 0.55)	0.24 (-0.17, 0.62)	< 0.001
TG (mg/dl)	136.61 ± 84.00	134.16 ± 83.05	149.57 ± 87.78	< 0.001
HDL-C (mg/dl)	46.40 ± 11.35	46.41 ± 11.36	46.36 ± 11.27	0.555
LDL-C (mg/dl)	101.86 ± 25.42	101.55 ± 25.19	103.51 ± 26.55	0.007
T-C (mg/dl)	185.11 ± 38.10	184.54 ± 37.64	188.13 ± 40.24	0.003
ALP (UI/L)	197.11 ± 61.02	194.46 ± 56.52	211.13 ± 79.29	< 0.001
AST (UI/L)	21.43 ± 8.95	21.47 ± 9.06	21.20 ± 8.36	0.325
ALT (UI/L)	24.70 ± 14.28	24.84 ± 14.53	23.94 ± 12.88	0.045
GGT (UI/L)	24.30 ± 18.89	23.86 ± 18.58	26.63 ± 20.30	< 0.001
Hypertension, n (%)	1351 (18.35)	411 (6.64)	940 (80.27)	< 0.001
Anti- Hypertension medications (%)	734 (23.20)	6 (0.29)	728 (65.41)	< 0.001
Dyslipidemia, n (%)	3085 (41.90)	2567 (41.46)	518 (44.24)	0.078
Anti- Dyslipidemia medications (%)	277 (10.14)	32 (1.96)	245 (22.31)	< 0.001
Diabetes, n (%)	623 (22.46)	360 (582)	263 (22.46)	< 0.001
Anti-diabetic medications (%)	393 (14.38)	184 (11.25)	209 (19.03)	< 0.001
MetS, n (%)	1891 (25.69)	1261 (20.37)	630 (53.80)	< 0.001

*BMI* Body mass index, *WHR* Waist hip ratio, *WC* Waist circumference, *BFM* Body fat mass, *PBF* Percent body fat, *VFA* Visceral fat area, *SLM *Skeletal muscle mass, *VAI* Visceral Adiposity Index, *AIP* Atherogenic index of plasma, *TG* Triglycerides, *HDL-C* high-density lipoprotein cholesterol, *LDL-C* Ligh-density lipoprotein cholesterol, *T- C* Total cholesterol, *FBS* Fasting blood sugar, *ALP* Alkaline phosphatase, *AST* Aspartate transaminase, *ALT* Alanine aminotransferase, *GGT* Gamma-glutamyl transferase, *CVDs* Cardiovascular diseases, *MetS* Metabolic syndrome

^*^Median (IQR)

### Basic Characteristics of Participants According to Tertiles of Visceral Adiposity Index (VAI) and Atherogenic Index of Plasma (AIP)

Table 2 shows the values of AIP and VAI expressed as tertile according to the cut-off points reported for them in the Methodology Section. Mean BMI in the first to third tertile of VAI was equal to 25.49 ± 4.11, 27.55 ± 3.57, and 28.11 ± 3.24 kg/m^2^, respectively (*P* < 0.001) in males; also BMI was increased significantly with the increase in VAI in females. Mean FBS in the first to third tertile of VAI was equal to 95.10 ± 29.01, 99.48 ± 30.83, and 107.86 ± 41.04 mg/dl, respectively (*P* < 0.001) in males; also FBS was increased significantly with the increase in VAI in females. There were significant differences in anthropometric indices including WC and WHR and lipid profile (TG, HDL-C, LDL, and TC) between VAI tertiles (*P* < 0.001) in males and females. Participants with higher VAI had significantly more hypertension, dyslipidemia, MetS, and CVDs compared to those with low VAI (Table 2).

**Table 2:** Baseline Characteristics according to tertiles of visceral adiposity index (VAI) and atherogenic index of plasma (AIP) among by sex

**Parameters**	**Tertiles of Visceral Adiposity Index**							
	**Male**				**Female**			
	T1 (*n*= 684)	T2 (*n*= 1228)	T3 (*n*= 1508)	*P*value*	T1 (*n*= 2453)	T2 (*n*= 1097)	T3 (*n*= 392)	*P*value*
Age (year)	46.78± 8.27	46.66± 7.82	46.01± 7.74	0.319	46.90± 8.43	48.74± 8.37	49.15± 8.61	<0.001
BMI (kg/m^2^)	25.49±4.11	27.55± 3.57	28.11± 3.24	<0.001	27.78± 4.96	29.62± 4.50	29.60± 3.90	<0.001
WHR	0.93± 0.07	0.95± 0.06	0.96± 0.05	<0.001	0.93± 0.06	0.95± 0.06	0.96± 0.05	<0.001
WC (cm)	94.14± 9.75	98.79± 8.72	100.11± 7.52	<0.001	96.10± 11.45	100.72± 9.87	101.35±9.35	<0.001
TG (mg/dl)	102.38± 35.10	195.15± 41.60	344.98± 100.53	<0.001	89.77± 28.37	159.37± 38.27	278.93± 83.89	<0.001
HDL-C (mg/dl)	45.87± 9.54	36.84± 6.40	32.82± 6.10	<0.001	53.97± 10.67	43.81± 8.10	38.49± 7.19	<0.001
LDL-C (mg/dl)	98.48± 24.46	106.38± 24.78	104.49± 23.71	<0.001	98.45± 24.46	108.41± 26.75	113.43± 28.10	<0.001
T-C (mg/dl)	176.60± 35.10	188.63± 36.43	198.11± 40.67	<0.001	182.04± 36.16	195.88± 36.96	207.60± 43.58	<0.001
FBS (mg/dl)	95.10± 29.01	99.48± 30.83	107.86± 41.04	<0.001	93.01± 24.06	100.89± 33.61	108.73± 40.33	<0.001
Hypertension, n (%)	314 (13.59)	132 (15.85)	49 (17.75)	<0.001	435 (17.73)	307 (27.99)	114 (29.08)	<0.001
Dyslipidemia, n (%)	743 (32.15)	761 (91.36)	276 (100)	<0.001	326 (13.29)	588 (53.60)	390 (99.74)	<0.001
Diabetes, n (%)	147 (6.36)	83 (9.96)	45 (16.30)	<0.001	137 (5.58)	131 (11.94)	80 (20.41)	<0.001
MetS, n (%)	418 (18.09)	320 (38.42)	111 (40.22)	<0.001	415 (16.92)	426 (38.83)	201 (51.28)	<0.001
CVD, n (%)	243 (10.51)	101 (12.12)	40 (14.49)	<0.001	403 (16.43)	279 (25.43)	105 (26.79)	<0.001
**Tertiles of Atherogenic index of plasma**								
	T1 (*n*= 1288)	T2 (*n*= 511)	T3 (*n*= 1621)	*P*value*	T1 (*n*=2144)	T2 (*n*= 564)	T3 (*n*= 1234)	*P*value*
Age (year)	47.03± 8.27	46.43± 8.34	46.50± 7.92	0.171	46.76± 8.44	48.62± 8.55	48.70±8.33	<0.001
BMI (kg/m^2^)	24.59± 3.93	26.21± 3.92	27.48± 3.73	<0.001	27.45± 4.81	29.89± 4.91	29.60± 4.37	<0.001
WHR	0.91± 0.06	0.94± 0.07	0.95± 0.06	<0.001	0.93± 0.06	0.96± 0.05	0.96± 0.05	<0.001
WC (cm)	92.69± 9.68	95.83± 9.61	98.16± 8.89	<0.001	95.76± 11.44	100.90± 10.73	100.19± 9.76	<0.001
TG (mg/dl)	80.12± 22.65	116.61± 19.56	204.55± 85.90	<0.001	84.67± 25.52	129.81± 22.04	202.30± 77.55	<0.001
HDL-C (mg/dl)	49.37± 9.85	42.45± 6.82	37.30± 7.04	<0.001	54.90± 10.70	47.30± 7.84	41.44± 7.92	<0.001
LDL-C (mg/dl)	94.46± 24.27	101.70± 22.33	105.74± 24.67	<0.001	97.46± 24.31	106.12± 23.94	110.27± 27.54	<0.001
T-C (mg/dl)	171.83± 34.70	179.48± 32.20	189.32± 37.54	<0.001	180.77± 36.19	192.20± 36.32	200.03± 41.80	<0.001
FBS (mg/dl)	93.52± 29.20	95.46± 27.96	100.55± 32.50	<0.001	92.14± 21.97	99.02± 34.62	103.77± 35.83	<0.001
Hypertension, n (%)	230 (17.86)	209 (40.90)	1341 (82.73)	<0.001	355 (16.56)	152 (26.95)	349 (28.28)	<0.001
Dyslipidemia, n (%)	108 (12.47)	424 (37.89)	1248 (86.97)	<0.001	246 (11.47)	154 (27.30)	905 (73.34)	<0.001
Diabetes, n (%)	66 (5.13)	33 (6.46)	176 (10.86)	<0.001	100 (4.66)	58 (10.28)	190 (15.40)	<0.001
MetS, n (%)	176 (13.66)	97 (18.98)	576 (35.53)	<0.001	346 (16.14)	151 (26.77)	545 (44.17)	<0.001
CVD, n (%)	125 (9.70)	58 (11.35)	201 (12.40)	<0.001	339 (15.81)	124 (21.99)	324 (26.26)	<0.001

*BMI* Body mass index, *WHR* Waist hip ratio, *WC* Waist circumference, *CVD* Cardiovascular diseases, *TG* Triglycerides, *HDL-C* high-density lipoprotein cholesterol, *LDL-C* Low-density lipoprotein cholesterol, *T- C* Total cholesterol, *FBS* Fasting blood sugar, *TG* Triglycerides, *T- C* Total cholesterol, *CVDs* Cardiovascular diseases, *MetS* Metabolic syndrome

Mean WHR in the first to third tertile of AIP was equal to 0.91 ± 0.06, 0.94 ± 0.07, and 0.95 ± 0.06, respectively (*P* < 0.001) in males. There were significant differences in anthropometric indices and lipid profile between AIP tertiles (*P* < 0.001). Also, participants with higher AIP had significantly more hypertension, dyslipidemia, MetS, and CVDs compared to those with low AIP (Table 2).

### The Association of AIP and VAI with Cardiovascular Diseases

Univariate logistic regression analysis showed that the increase in AIP level was associated with an increased risk of CVDs. Risk of CVDs in the second and third tertiles of AIP was 1.30 (95 % CI: 1.10 1.57) and 1.44 (95 % CI: 1.26, 1.65) times higher than the first tertile, respectively. In multiple logistic regression analysis and after adjusting for age and sex, an increase in AIP and VAI levels was significantly associated with an increased risk of CVDs (Model2). Moreover, analysis results of Model 3 showed that after adjusting for age, sex, BMI, physical activity, hypertension, and dyslipidemia, risk of CVDs was increased in the third tertile of AIP by 1.32 (95 % CI: 1.03, 1.69) times compared to the first tertile; and in the third tertile, VAI was increased by 1.48 (95 % CI: 1.12, 1.97) times than the first tertile (Table 3).

**Table 3:** Association of atherogenic index of plasma and visceral adiposity index with cardiovascular diseases (logistic regression analysis)

Variables	Model 1		Model 2		Model 3		Model 4	
	OR (95% CI)	***P*** value	OR (95% CI)	***P*** value	OR (95% CI)	***P*** value	OR (95% CI)	***P*** value
**AIP :** T1	1.00 (Reference)		1.00 (Reference)		1.00 (Reference)		1.00 (Reference)	
T2	1.30 (1.10 1.57)	0.005	1.29 (1.10, 1.57)	0.012	1.01 (1.02, 1.45)	0.107	1.16 (0.94, 1.43)	0.155
T3	1.44 (1.26, 1.65)	<0.001	1.60 (1.38, 1.86)	<0.001	1.32 (1.03, 1.69)	<0.001	1.15 (0.99, 1.35)	0.073
**VAI :** T1	1.00 (Reference)		1.00 (Reference)		1.00 (Reference)		1.00 (Reference)	
T2	1.55 (1.32, 1.82)	<0.001	1.54 (1.30, 1.83)	<0.001	1.08 (0.86, 1.37)	0.467	1.38 (1.16, 1.65)	<0.001
T3	1.64 (1.40, 1.93)	<0.001	1.85 (1.56, 2.19)	<0.001	1.48 (1.12, 1.97)	0.006	1.25 (1.04, 1.50)	0.019

Model 1: Unadjusted; Model 2: Adjusted for age and sex, Model 3: Adjusted for age, sex, BMI, physical activity, hypertension and dyslipidemia; Model 3: Adjusted for age, sex, metabolic syndrome

*AIP* Atherogenic index of plasma, *VAI* Visceral Adiposity Index, *T* tertile

**Fig. 2:**
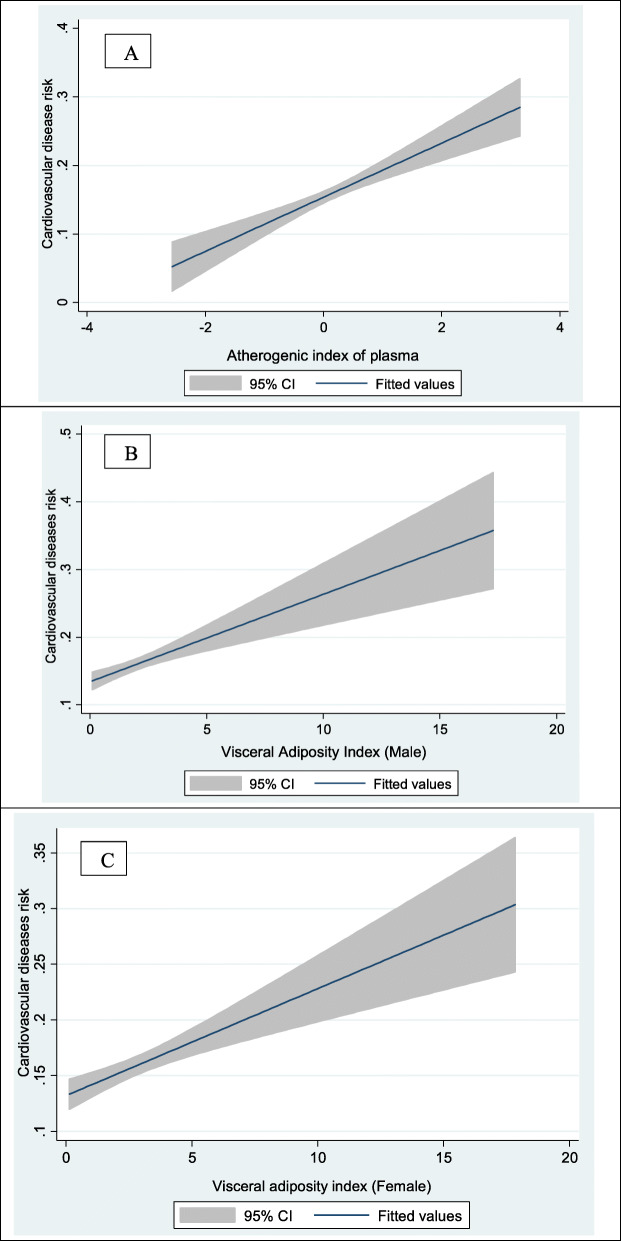
Associations atherogenic index of plasma (**A**), visceral adiposity index in male (**B**) and visceral adiposity index in female (**C**) with cardiovascular diseases

Figure 2 schematically shows the increased risk of CVDs with the increase in AIP and VAI levels in participants.

## Discussion

Findings of this study showed a positive association between AIP and VAI with risk of CVDs in adults, and this association remained significant after adjusting for potential confounders. In addition, males and females with higher VAI and AIP had significantly more hypertension, dyslipidemia, MetS, and CVDs compared to those with low levels of VAI and AIP. Higher levels of anthropometric indices, lipid profile, and FBS were also associated with higher levels of VAI and AIP.

Mean levels of AIP and VAI in this study were somewhat higher than other studies [20–22], which could be due to the studied age group (between 35 and 65 years old) and high prevalence of obesity and overweight in participants. Moreover, mean level of VAI was higher in female participants compared to males; higher level of VAI was associated with higher prevalence of CVDs in both males (first tertile: 20 % vs. third tertile: 39.71 %) and females (first tertile: 5.98 % vs. third tertile: 65.33 %). However, in this study, although males҆ BMI was higher compared to the females, the females҆ VAI was higher than the males.

Findings of this study showed that risk of CVDs was increased in the third tertile of VAI (OR: 1.85, *P* < 0.001) compared to the first tertile. The association between VAI, heart disease and its risk factors has been reported in the previous studies. For example, results of a cohort study conducted in Greece showed that VAI was independently associated with CVDs [26]. Moreover, it was indicated that participants with higher VAI had significantly more hypertension, dyslipidemia, T2DM, and MetS compared to those with low level of VAI. Similar to current findings, results of a meta-analysis study done in 2019 demonstrated that VAI can be an independent predictor of T2DM in Asian populations [11]. In addition, predictive power of VAI using receiver operating characteristic (ROC) curve analysis has shown accuracy and reliability of VAI for predicting T2DM, hypertension, and MetS [10, 12, 14]. Overall, this finding is important because these factors are risk factors for CVDs; and CVDs can be prevented by controlling these risk factors.

Findings of the present study demonstrated that high levels of anthropometric indices (BMI, WHR, and WC) were associated with higher risk of CVDs. BMI was equal to 27.2 and 28.7 kg/m^2^ in non-CVDs and CVDs groups, respectively and WHR was equal to 0.93 and 0.95 among non-CVDs and CVDs groups, respectively. Zhu et al., presented that an increase in levels of anthropometric indices (BMI and WC) and liver enzymes (ALT and GGT) and LDL-C was associated with an increase in AIP levels [29], which is consistent with the findings of this study. Moreover, results of another study indicated that mean VFA in patients with CVDs was significantly higher compared to patients without CVDs (118.18 vs. 141.71 cm^2^), which is consistent with the findings of a study by Bo et al., [30]. Mechanism of this association can be expressed as follows: Visceral fat is mainly depleted by portal venous system and then, is discharged into the liver leading to insulin resistance. Besides, excess free fatty acids (FFA) may cause enhancement of lipid synthesis and gluconeogenesis, as well as insulin resistance, resulting in hyperlipidemia, glucose intolerance, hypertension, and finally atherosclerosis [30].

This study showed that the odds of CVDs were increased in the third tertile of AIP (OR = 1.60, *P* 0.001) compared to the first tertile, which can be a good marker to predict CVDs. A positive association between AIP and CVDs has already been observed in different populations, such as postmenopausal women and staff [19, 30]. Moreover, AIP is positively associated with non-alcoholic fatty liver disease (NAFLD), advanced subclinical coronary artery disease (CAD), ischemic stroke, atherosclerosis, MetS, and obesity; and is a useful and reliable marker for predicting these diseases [29, 31–34]. Results of a study on Malaysian population showed that among lipid profile indices, AIP was more positively correlated with TG and was more negatively correlated with HDL-C, thus AIP was considered to be the strongest marker in predicting risk of CVDs among the other indices [30]. Other research has shown that AIP is the most sensitive marker compared to other atherogenic indices including (TC/HDL-C), (LDL-C/HDL-C), and atherogenic coefficient (TC-HDL-C/HDL-C) [35, 36]. Isolated elevation in TG level increases risk of CVDs but these effects may be balanced by cardio-protective lipoprotein of HDL-C [37].

Following industrialization of societies and rapid growth of urbanization, physical activity has been reduced, which has led to an increase in general and central obesity, and the increased prevalence of pre-diabetes, T2DM, hypercholesterolemia, hypertension, and MetS [38–40]. Considering limitations of older indices, such as BMI and WC- mentioned in the previous studies [41–43] - as well as high prevalence of morbidity and mortality related to CVDs, and the need for a suitable tool to screen them; VAI and AIP can be a useful, inexpensive, and accurate tool for predicting NCDs, especially CVDs [28].

### Strengths and limitations of the study

The most important strength of this study was the use of baseline data of RaNCD prospective study. This is the first study about the association of AIP and VAI with CVDs on a large population with Kurdish ethnicity. Large sample size was one of positive points of this study. The main limitation of this study was its cross-sectional nature, limiting causal inference of the observed associations. Moreover, findings of this study cannot be generalized to all age groups and other ethnicities, because this study was performed only on adult people of Kurdish ethnicity living in western part of Iran. Therefore, further studies are suggested to be conducted in different regions and population groups.

## Conclusions

Findings of this study demonstrated a positive association between AIP and VAI with risk of CVDs in adults, and this association remained significant after adjusting for potential confounders. Therefore, AIP and VAI can be used as a low-cost and convenient tool for early detection of CVDs in populations.

## Data Availability

The data analyzed in the study are available from the corresponding author on reasonable request.

## Abbreviations

AIP: Atherogenic index of plasma
BMI: Body mass index
CVDs: Cardiovascular diseases
CAD: Coronary artery disease
FFA: Free fatty acids
FBS: Fasting blood sugar
NCDs: Non-Communicable disease
HDL-C: High density lipoprotein
MI: Myocardial infraction
IHD: Ischemic heart disease
ALP: Alkaline phosphatase
AST: Aspartate transaminase
ALT: Alanine aminotransferase
GGT: Gamma-glutamyl transferase
MetS: Metabolic syndrome
MET: Metabolic equivalents
PBF: Percent body fat
VFA: Visceral fat area
SLM: Skeletal muscle mass
VAI: Visceral Adiposity Index
NAFLD: Non-alcoholic fatty liver disease
RaNCD: Ravansar non-communicable diseases
PERSIAN: Prospective epidemiologic research of IRAN
VLDL: Very-low-density lipoprotein
VAI: Visceral Adiposity index
LDL-C: Low density lipoprotein
TG: Triglyceride
WC: Waist circumference
WHR: Waist to hip ratio
VAT: Visceral adipose tissue
SAT: Subcutaneous adipose tissue
CI: Confidence interval
T2DM: Type 2 diabetes mellitus
SBP: Systolic blood pressure
DBP: Diastolic blood pressure
BIA: Bio-Impedance Analyzer
IDF: International Diabetes Federation
